# USB capsule endoscope for retrograde imaging of the esophagus

**DOI:** 10.1117/1.JBO.25.10.106002

**Published:** 2020-10-19

**Authors:** Ivan Martincek, Peter Banovcin, Matej Goraus, Martin Duricek

**Affiliations:** a University of Zilina, Department of Physics, Faculty of Electrical Engineering and Information Technology, Zilina, Slovakia; b Comenius University in Bratislava, Department of Gastroenterology, Jessenius Faculty of Medicine in Martin, Martin, Slovakia

**Keywords:** capsule endoscope, retrograde imaging, esophagogastroduodenoscopy

## Abstract

**Significance:** Endoscopes represent electro-optical devices that are used to visualize internal body cavities. The specialized endoscopic procedure of the upper gastrointestinal tract from the esophagus down to the duodenum is called an esophagogastroduodenoscopy.

**Aim:** We bring our newly developed capsule endoscopy device as a promising alternative diagnostic method for visualization of the upper gastrointestinal tract.

**Approach:** Capsule endoscopy has become an attractive method that uses a tiny wireless camera to take pictures of the digestive tract. Existing esophageal capsule endoscopy does not allow a retrograde view of the esophagus while retrograde scanning can provide information on the esophageal pathology.

**Results:** In comparison to the existing esophageal capsule endoscopy, our system is much simpler and cheaper due to the need for fewer electronic devices. Moreover, its use is not limited by the capacity of the batteries used by existing capsule endoscopes. The new esophageal endoscopic system was created by combining the universal serial bus (USB) endoscope module with the thin power wires that are routed through the USB port to the computer.

**Conclusions:** The endoscope was tested on a volunteer without any side effects such as nausea, belching, and general discomfort. The examination of the patient is performed in a sitting position and the patient discomfort during the examination is minimal so it can be performed without anesthesia.

## 1 Introduction

Endoscopes are electro-optical devices that are used to visualize the internal body cavities. The specialized endoscopic procedure of the upper gastrointestinal tract from the esophagus down to the duodenum is called an esophagogastroduodenoscopy (EGD). EGD visualizes the upper part of the gastrointestinal tract. During the EGD, a flexible hose with a small video camera, lighting, and other special components is inserted through the patient’s mouth or nose. The diameter of the endoscope hose ranges from ∼6 to 10 mm. EGD is usually done when the patient is in the left lateral position, and it can be performed without sedation, using only topical anesthesia, or under sedation, which generally results in better patient tolerance and comfort.

The examination of the patient’s esophagus is usually performed using conventional EGD and is generally considered to be an optimal method for the visualization of the esophageal mucosa.^1^ However, EGD also has some disadvantages. It can be annoying and can cause nausea, gagging, choking, and overall patient discomfort. These disadvantages can cause the patient to reject EGD. Therefore, other alternative diagnostic methods for the examination of the esophagus are being developed.

Recently, capsule endoscopy has become an attractive method, which has been developed primarily for the examination of the small intestine.^2^ Since its inception, several wireless types of swallowed imaging capsules have been developed that allow various parts of the digestive tract to be imaged.^3^^–^^5^ Although white light imaging is used as the fundamental technology in clinical capsule endoscopy at present, other sensing modalities for capsule endoscopy are being developed, such as nonwhite light imaging,^6^^,^^7^ fluorescent imaging,^8^^,^^9^ optical coherence tomography, which uses a tethered capsule assembly,^10^^–^^12^ and more.^13^

Standard capsule endoscopy is a procedure that uses a tiny wireless camera to take pictures of the digestive tract. A capsule endoscopy camera sits inside a small capsule that is swallowed. As the capsule travels through the digestive tract, the camera takes pictures that are transmitted to a data recorder, which the patient wears around the waist. The capsule endoscope system generally consists of three components: wireless capsule, data recorder, and computer with the software for analysis of the pictures.^14^

For the esophagus examination, a commercial wireless capsule endoscope system PillCam ESO was developed, which allows examining the esophagus during a few-minutes procedure. During the examination, a capsule with dimensions of $26\times 11\;{\mathrm{mm}}^{2}$ is swallowed and within a few minutes the patient is supine, raised to 30°, raised to 60°, and followed by an upright position.^15^

For the better positioning of the wireless capsule in the esophagus and for time prolonging of its passing through the esophagus, modifications to the capsule endoscope are under development. These modifications are based on the wireless capsule’s attachment to strings, which control the position of the capsule in the esophagus.^16^ The stringed esophageal wireless capsule endoscopy is considered to improve the operability and diagnostic yield in the identification of esophageal pathology.^17^ In addition, the capability of the wireless device for providing the retrograde view of certain anatomical structures of the esophagus [e.g., the upper and lower esophageal sphincter (LES)] would be desirable for both clinical and research purposes.

According to our experimental experiences, the stringed esophageal wireless capsule endoscopy does not allow retrograde views of the esophagus. If the capsule is attached to the strings as is described in Ref. 17, the mucus is trapped between the strings during the retrograde scanning and it makes capturing an image impossible. The retrograde scanning can provide new information on the esophageal pathology. Our research team has, therefore, developed a new alternative with and without the string for esophageal wireless capsule endoscopy. The new esophageal endoscopic system was created by combining the universal serial bus (USB) endoscope module with the thin power wires that are routed through the USB port to the computer. In comparison to the existing esophageal capsule endoscopy, our system is much simpler and cheaper due to the need for fewer electronic devices. Moreover, its use is not limited by the capacity of the batteries used by existing capsule endoscopes. In our endoscope system, the video camera captures the retrograde image of the esophagus with real-time viewing and its position in the esophagus is controlled by the length of the swallowed cable. The examination of the patient is performed in a sitting position, and the patient discomfort during the examination is minimal so it can be performed without anesthesia.

In the next part of this paper, we describe the fabrication method of the new esophageal endoscopic system and its technical parameters, and we demonstrate its imaging properties during the examination of the esophagus of a volunteer.

## 2 Endoscope Manufacturing Procedure

The endoscope was made from a commercial USB endoscope camera module with six light-emitting diodes (LEDs). The USB module was connected to a USB cable and a computer using thin power cables that were inserted into a plastic tube with an outer diameter of 1.2 mm. During the examination of the esophagus, the patient swallows the endoscopic module, which remains hanging on the plastic tube in the esophagus. The doctor adjusts the position of the endoscopic capsule in the esophagus by adjusting the length of the swallowed plastic tube.

For the fabrication of the endoscopic capsule, we used a USB endoscope module with the parameters given in Table 1.

**Table 1:** Endoscope module parameters.

Segment	Description
Sensor	1/9 in. CMOS
Resolution	640×480
Frame rate	30 fps
View angle	70 deg
Power supply	5V DC via USB
Light	6 white LED
Brightness	Auto
Focus type	Autofocus
Video format	AVI
Depth of focus	4 cm – infinity
Dimension	$20\times 6\;{\mathrm{mm}}^{2}$

As the power wires for the endoscopic module, the three insulated copper wires with a diameter of 0.25 mm and a length of 70 cm were used. The video signal from the module was let out using a coaxial cable (Molex coaxial cables 42 AWG PFA, 50 Ω) also with a length of 70 cm. All these wires were inserted into a flexible polyvinyl chloride (PVC) tube with an outer diameter of 1.2 mm and wall thickness of 0.2 mm. The ends of the wires were soldered to the module and together with the tube, the part of the wires was attached to the endoscopic module by a nylon thread. The wires were led out from the module in the tube along its edge on the module side with an optical input. Such an output of the wires from the capsule ensured very small trapping of the mucus on the sensor lens.

**Fig. 1:**
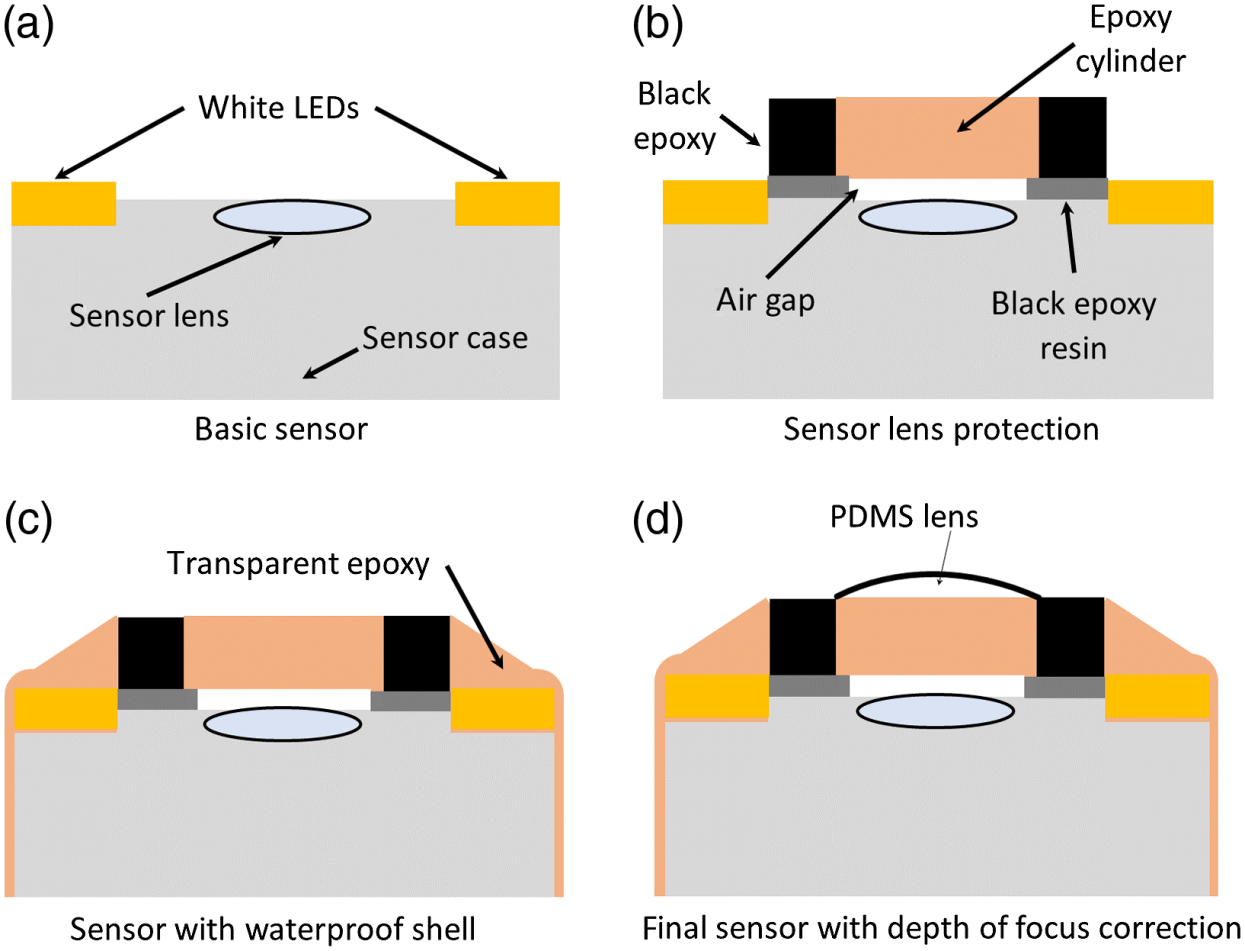
Water-resistant modification of the sensing part of the USB endoscope camera module suppressing the light input from the LED to the sensor lens: (a) basic sensor without modification, (b) additional protection of sensor lens with epoxy cylinder and black epoxy shield, (c) application of transparent epoxy to achieve water resistance of sensor, and (d) PDMS lens application to change the depth of focus.

Before the water-resistant module preparation, we modified the optical input of the endoscope module [Fig. 1(a)]. Since the lighting LEDs of the module are situated near the sensor lens, the scattered light from the LEDs reaches the image sensor and illuminates the scanned image. To suppress this effect, the epoxy cylinder with a diameter of 3 mm and a height of 1 mm was applied on the sensor lens. We applied a black epoxy layer with a thickness of 1 mm to the edge of the cylinder, which was firmly attached to the cylinder. The epoxy cylinder with a black epoxy layer was air-tight attached with black epoxy resin to the plastic enclosure of the endoscopic module such that an air gap with a thickness <0.5  mm was formed between the epoxy cylinder and the sensor lens. The black epoxy layer was situated between the lighting LEDs and the sensor lens, preventing illumination from the lighting LEDs from leaking to the sensor lens [Fig. 1(b)].

In the final modification, the module with the wires, tube, and epoxy cylinder with a black epoxy edge was encapsulated with a transparent epoxy resin to form a water-resistant retrograde endoscopic capsule [Fig. 1(c)]. For fabrication of all epoxy parts of the endoscope, a medical two-component resin Loctite EA M–31CL was used. A fabrication process of water-resistant modification of the sensing part of the USB endoscope camera module used to suppress the light input from the LED to the sensor lens is shown in Fig. 1.

The used capsule module has a depth of focus from 40 mm to infinity. The capsule endoscope usually has a depth of focus ranging from 0 to 30 mm.^18^ To adjust a suitable depth of focus of the sensor lens and reduce the wettability of the optical input of the module in a humid environment, we applied epoxy cylinder and a cylindrical polydimethylsiloxane (PDMS) planoconvex lens layer [Fig. 1(d)]. PDMS was prepared from Sylgard 184 elastomer. PDMS is a transparent, hydrophobic, flexible, and rapidly prototyped elastomeric material that is often used in microfluidics. Within tens of minutes after immersion, PDMS is well resistant to both water and hydrochloric acid,^19^ which are suitable properties in esophagus examinations.

A PDMS lens was prepared experimentally. We applied liquid PDMS to an epoxy cylinder with a diameter of 3 mm located above the sensor lens. The liquid PDMS formed a cylindrical planoconvex lens on the epoxy cylinder. The thickness and curvature of this lens depended on the amount of applied PDMS. By this process, we changed the depth of focus of the sensor module optical system. During the application of liquid PDMS, we checked the depth of focus by real-time imaging from the sensor module. When we adjusted the depth of focus approximately from 1.5 to 4 cm, we cured the liquid PDMS. Due to the surface tension and wetting phenomena, the liquid PDMS forms a planoconvex lens on the epoxy cylinder. The radius of curvature was 7.4 mm and we used a volume of $0.63\;{\mathrm{mm}}^{3}$ of liquid PDMS to prepare the lens.

**Fig. 2:**
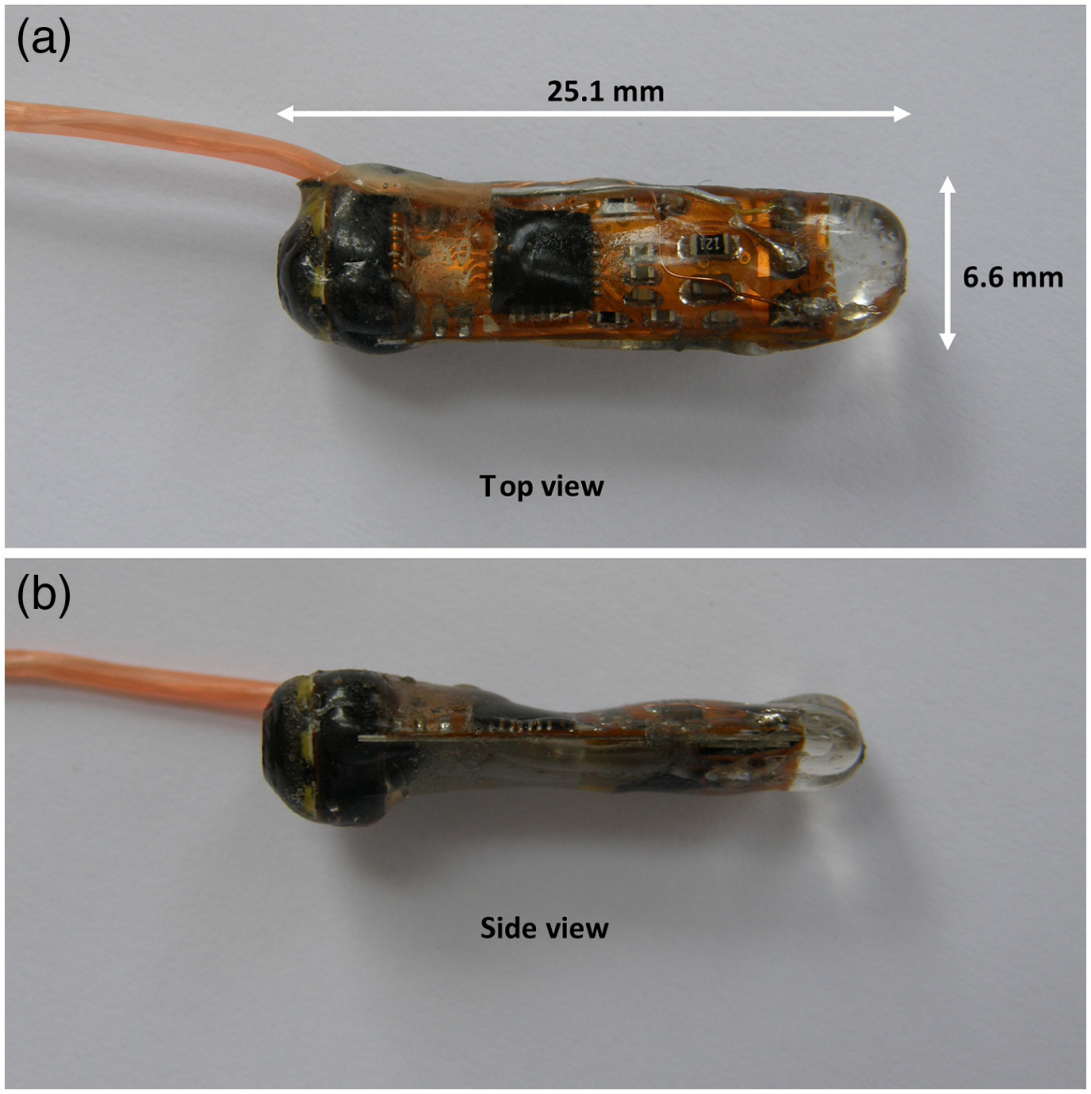
Finished water-resistant retrograde USB endoscope camera module: (a) top view and (b) side view.

After heat curing, the prepared PDMS lens had a center thickness of 0.165 mm. Although PDMS is permeable to water vapor in the long term, which may reduce the optical quality of PDMS, during the endoscopic examination in the upper part of the gastrointestinal tract, the PDMS lens retained suitable optical properties. The detailed view of the prepared water-resistant module is shown in Fig. 2.

**Fig. 3:**
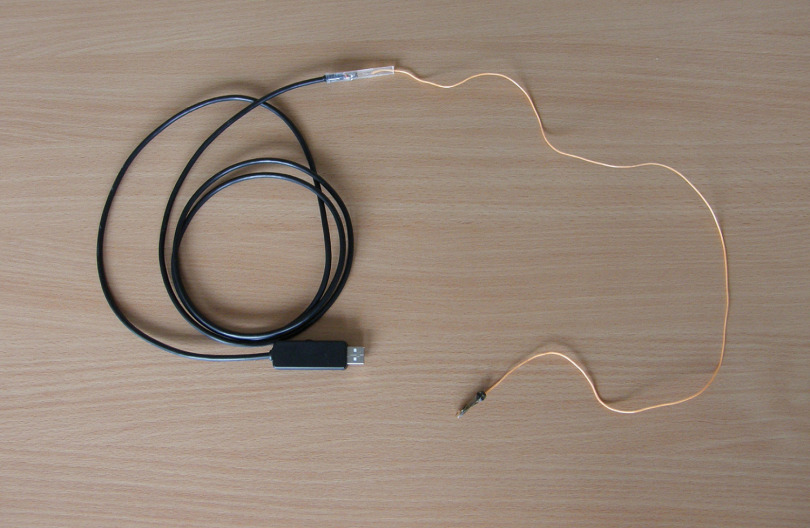
Finished USB endoscope allowing retrograde scanning of the upper part of the digestive tube.

Finally, the PVC tube with the power wires of the endoscopic module was firmly bonded by epoxy resin to the USB cable that plugs in the USB port of the computer. The picture of the whole prepared USB endoscope is shown in Fig. 3.

## 3 Endoscope Testing

**Fig. 4:**
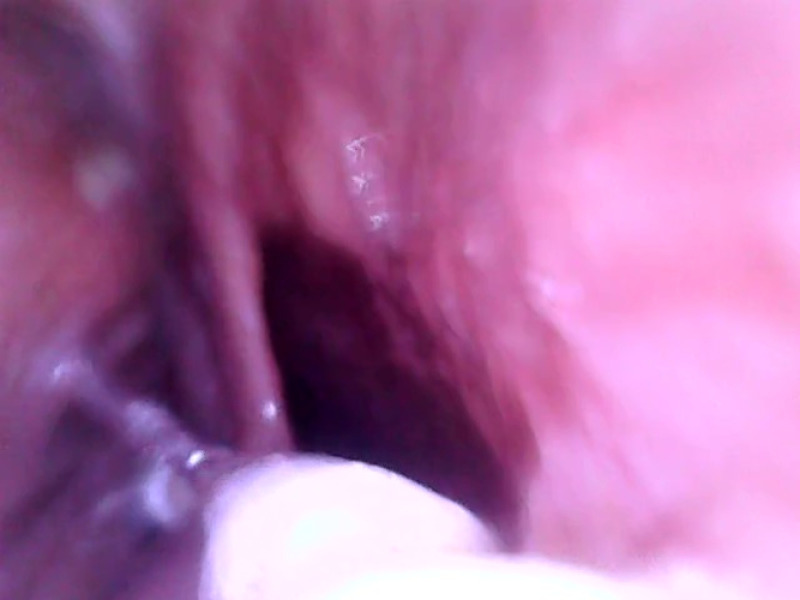
Oral cavity: posterior pharyngeal wall on the right side, the epiglottis on the left.

**Fig. 5:**
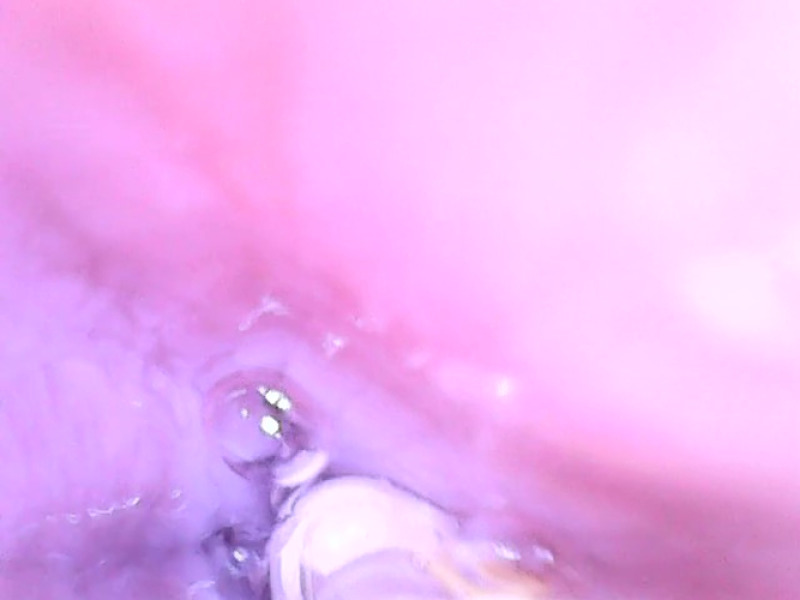
Proximal esophagus: the closing of the upper esophageal sphincter and the surrounding mucosa of the upper esophagus.

**Fig. 6:**
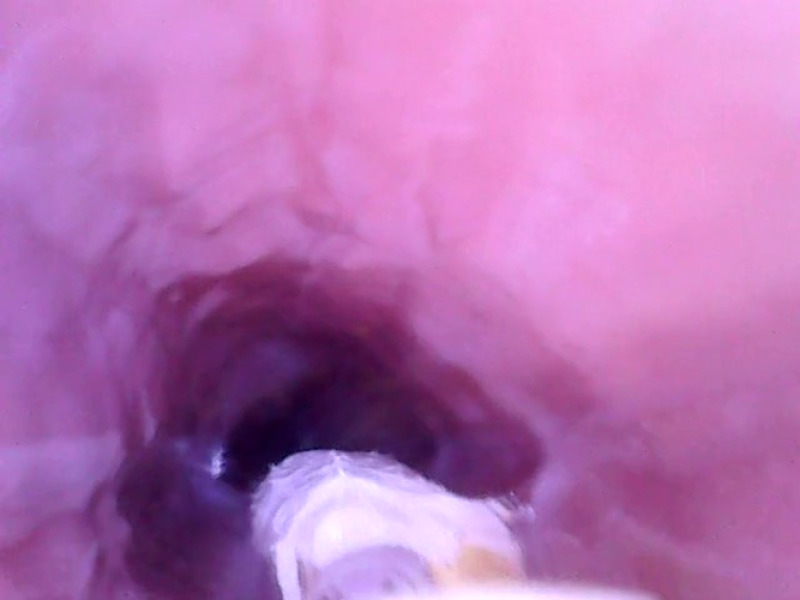
Midesophageal lumen and the esophageal mucosa.

**Fig. 7:**
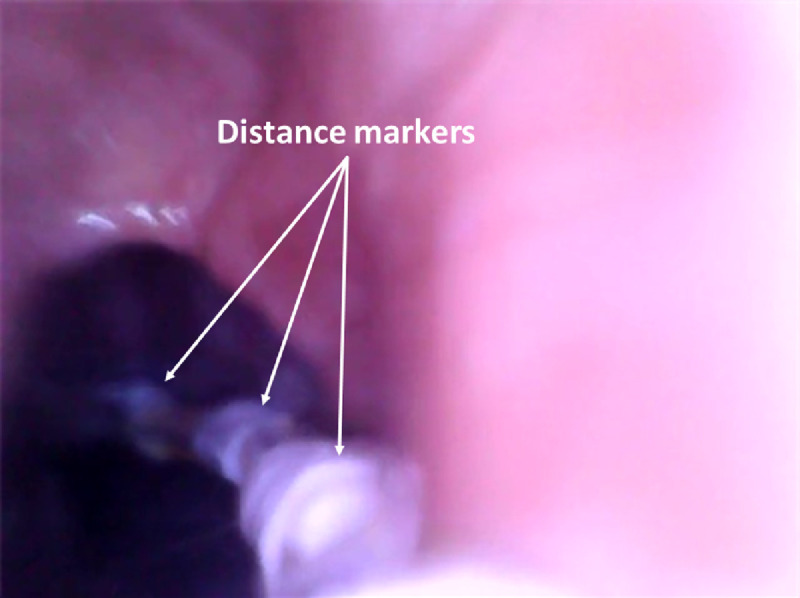
Stomach: below the LES—the dark part represents the LES just about to close, the surrounding mucosa represents the gastric fundus. Distance markers with a spacing of 2 cm are attached to the power wires. During the examination, they helped us determine the distance at which the capsule is located inside the stomach.

**Fig. 8:**
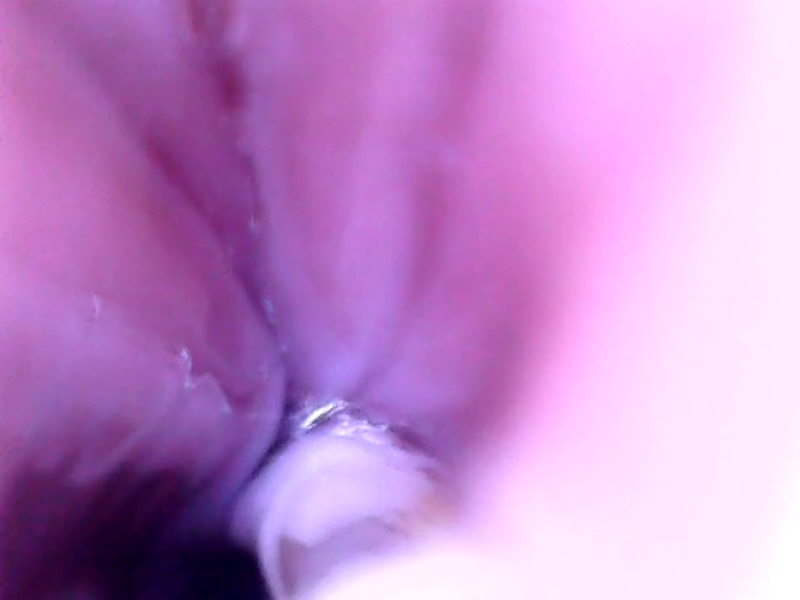
Gastric folds of the lesser curvature of the stomach.

**Fig. 9:**
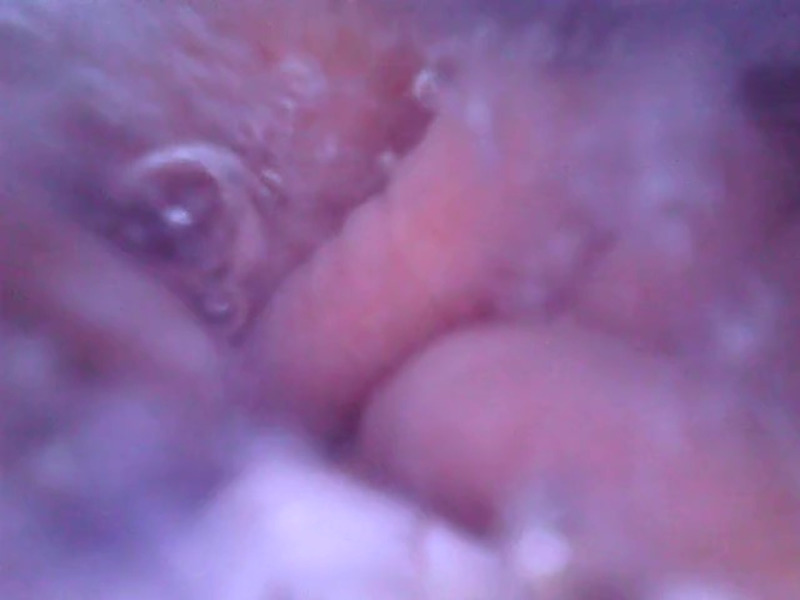
Gastric folds on the greater curvature with the clear gastric juice.

The finished endoscope was tested by a volunteer without any gastrointestinal symptoms. After overnight fasting, the volunteer swallowed the endoscope capsule in a sitting position and washed it down with 1 dl of water. The capsule was freely hanging in the esophagus on a thin PVC cable. During the whole examination, the volunteer remained in a sitting position. The position of the capsule in the esophagus was controlled by the length of the swallowed cable. The volunteer felt a very little discomfort and he could communicate verbally during the whole examination. The endoscope testing took 15 min and a continuous live video from the entire testing was made. During the examination, the endoscope capsule was moved from the oral cavity to the stomach, while the retrograde image was continuously captured. When the endoscope capsule reached the stomach, the volunteer drank another 2 dl of water to display a partially filled stomach. At the end of the examination, the endoscopic capsule was removed from the esophagus by pulling the PVC cable. Here, we provide unique retrograde images of the esophagus and stomach obtained during the examination (Figs. 4–9).

Figures 4–9 show selected parts of the retrograde image of the upper part of the gastrointestinal tract from the oral cavity to the stomach, which was obtained during the mentioned examination. During the endoscope testing, it was possible to observe the peristaltic contraction of the esophagus in various places due to the possibility of adjusting the position to different parts of the esophagus by a lead-in cable. Since the thickness of the lead-in cable was only 1.2 mm, the physiological activity of the esophagus was just minimally influenced. Figures 4–9 also document that the imaging properties of the prepared capsule endoscope are comparable to the commercial capsule endoscope systems.^20^

## 4 Conclusion

We consider our newly developed capsule endoscopy device a promising alternative diagnostic method for visualization of the esophagus. As seen above, it is capable of obtaining high-quality images of the mucosa of the upper gastrointestinal tract without the need for sedation, and without the discomfort and risks of conventional upper endoscopy. Moreover, a unique endoscopic view of certain anatomical structures is provided that cannot be acquired by conventional EGD. Although the conventional EGD represents the gold standard and the preferred endoscopic techniques for the study of the esophagus, some esophageal diseases may be detected using a capsule device.^20^^,^^21^

In this paper, we describe the fabrication method, properties, and results from the testing of the new capsule endoscope for retrograde imaging of the esophagus. By precise design of the optical input of the endoscopic module, we were able to suppress the effects of the internal light reflections, which are a common problem when lighting and imaging equipment are situated under the same dome. The wettability of the input optics of the module was reduced by applying a PDMS lens on the optical input of the module and the positioning control of the capsule in the esophagus was ensured by placing the lead-in wires into a thin PVC tube.

The prepared endoscope was tested on a volunteer without sedation and during the 15-min test the retrograde image of the esophagus and the upper part of the stomach was taken. Although the endoscopic capsule was hanging on a plastic tube, the endoscope was tested on a volunteer without any side effects such as nausea, belching, and general discomfort. The volunteer tolerated the endoscope testing very well. We believe that the described new type of USB capsule endoscope may be a suitable alternative to existing capsule endoscopes and may provide new possibilities for imaging of the esophagus and for diagnosing its diseases.
